# Reproduction affects immune defenses in the guinea pig even under ad libitum food

**DOI:** 10.1371/journal.pone.0230081

**Published:** 2020-03-16

**Authors:** Fritz Trillmich, Anja Guenther, Manuela Jäckel, Gábor Á. Czirják

**Affiliations:** 1 Department of Animal Behaviour, University Bielefeld, Bielefeld, Germany; 2 Max-Planck Institute for Evolutionary Biology, Evolutionary Genetics, Plön, Germany; 3 Department of Wildlife Diseases, Leibniz Institute for Zoo and Wildlife Research, Berlin, Germany; Central South University, The Third Xiang Ya Hospital, CHINA

## Abstract

Reproduction is one of the costliest processes in the life of an animal. Life history theory assumes that when resources are limiting allocation to reproduction will reduce allocation to other essential processes thereby inducing costs of reproduction. The immune system is vital for survival. If reproduction reduces investment in immune function, this could increase the risk of disease, morbidity and mortality. We here test in the guinea pig, if even under *ad libitum* food conditions, pregnancy and lactation reduce the activity of the adaptive and innate immune system compared to the reaction of non-reproducing animals. In response to a challenge with keyhole limpet haemocyanin the antibody-mediated adaptive immunity during (pregnancy and) lactation was reduced. Pregnant and lactating females showed higher levels of bacterial killing activity, an integrated measure of innate immunity, than non-reproducing females. However, two major effectors of the innate immunity, the natural antibody and the complement of pregnant and lactating females showed lower levels than in non-reproducing females. Pregnant and lactating females did not differ significantly in the expressed levels of innate immunity. Our results indicate that changes in the immune response during reproduction are physiological adjustments to predictable allocation problems, because they happen even under *ad libitum* food availability.

## Introduction

The demographic theory of optimal reproductive tactics [1] assumes that resources available to an individual are limited. As a consequence, selection acts on an animal's allocation decisions to create a balance between reproductive effort and self-maintenance in such a way that a maximal reproductive value results [1–3]. Given that reproductive effort can reduce further life expectancy [4] or future reproductive success [5] the question arises, which mechanisms the animals may use to balance increased expenses due to reproduction [6].

Increasing allocation for reproduction may lead to higher short-term fitness, but may reduce long-term survival through reduced expenditure on physiological maintenance and repair processes. One aspect that has received much attention is immune responses, which by defending the organism against micro- and macro-parasites constitute a major aspect of self-maintenance [7,8]. Negative effects of reproductive effort on aspects of immunity have been investigated intensively in birds [7,9,10]. Reproductive effort was shown to reduce the antibody response [11–13]. Similar effects have been documented in tree lizards (*Urosaurus ornatus*) and were shown to correlate with resource abundance [14]. In breeding female American kestrels (*Falco sparverius*), blood parasite levels correlated negatively with leucocyte numbers and antibody titres [15]. Similarly, breeding great tits (*Parus major*) appeared more susceptible to blood parasites (*Haematozoa* sp.) than non-breeding tits and in males susceptibility increased with increasing brood size and paternal feeding effort [16]. Weakening the immune system may in addition to endangering an animal's survival, also reduce the probability of successful reproduction by leading to death and abortion of fetuses [8].

Reproductive status has been shown to influence immunity [14,17,18]. In rats, lactating females proved less able to defend against a parasite infection than non-lactating females [19]. In spotted hyenas (*Crocuta crocuta*) lactating females have a higher incidence and burden of hookworm infection [20] and accordingly they show lower immune defenses compared to pregnant females [21]. Similar studies in ungulates and rabbits have shown increased infection rate during lactation [22–26]. Experimental reduction of litter size [27] as well as improved nutrition during lactation [28,29] improved immune defenses. Recently, Martin et al. [30] have reviewed and emphasized the existence of trade-offs between reproduction and immune function (see also [8]).

However, trade-offs may also occur within the immune system itself [30]. The immune system consists of two arms, the adaptive and the innate system, both with cellular and humoral effectors [31]. The adaptive arm is slower and presumably costlier to develop, but has the advantage of providing immunological memory and a specific, quick response during re-infection. In contrast, the innate system is unspecific, much quicker to respond than adaptive mechanisms [32] and its development is less costly than that of the adaptive immune system [30]. Although innate host defense is crucial for controlling an infection, it is often insufficient for complete pathogen clearance. In situations where energy becomes limiting, like seasonal food shortages or reproductive episodes, it has been shown that the two arms of the immune system may be differentially up- or down-regulated [30].

Such effects may be tested particularly well in mammals producing precocial young, that gestate significantly longer than altricial mammals and bear much larger young relative to maternal mass. These aspects make gestation particularly costly. Guinea pigs (*Cavia aperea* f. *porcellus*) belong to the most extremely precocial mammals [33,34]. Females produce about 40% of their own body mass during a pregnancy of 68 days and bear only 3–4 offspring [35,36]. Females are known to transfer antibodies to their offspring during gestation [37,38]. Young are born fully furred, independently able to thermoregulate, have a well-developed immune system [38] and show remarkable locomotor abilities [39]. Energy transfer is more efficient during pregnancy than during lactation [40] but still requires a 16% increase in energy intake above non-reproducing level [35]. The relative increase in energy demand reaches 92% above non-reproductive level during lactation [41]. Female metabolizable energy intake is increased to 2.4 times BMR level during pregnancy and to 3.7 times BMR level during lactation [35]. Thus lactation–as in other mammals [42–45]—represents the most energy demanding phase in the life cycle of the guinea pig despite the fact that soon after birth young begin to contribute substantially to their own energy demands by independent feeding on solid food [41]. Given that females come into estrus and copulate immediately post-partum they need strong immune defenses to avoid infection, e.g. directly and sexually transmitted diseases. This implies a necessity to invest in immune defense despite the energetic burden of reproduction. The great additional demand of energy and nutrients due to reproduction combined with the need to protect against post-partum infection make guinea pigs particularly suited to look for trade-offs between reproduction and immune function and potential trade-offs within the immune system. Moreover, it is unclear what triggers these trade-offs: Are they only occurring in response to food shortage or do they represent an inbuilt response of the physiological system to preempt potential resource shortages?

We tested the following predictions of the hypothesis that increased resource demands during reproduction will decrease the immunity. (1) In Experiment I we tested whether reproducing female guinea pigs show a lower antibody-mediated adaptive immune response to keyhole limpet hemocyanin (KLH) than non-reproducing animals. Since lactation is more energy demanding than pregnancy, we expected that this reduction should be most strongly expressed during lactation. (2) There may also be a shift in defense between the adaptive and the innate immune system. Therefore, in Experiment II, we compared the expression of these two arms of the immune system in non-reproducing, pregnant and lactating guinea pigs to test for the possibility of a shift in allocation within the immune system. Besides measuring the KLH-specific antibody levels, to get an overview on the innate immune system, we measured the bacterial killing activity (BKA) of plasma against *E*. *coli*, an integrated measure to characterize the overall functionality of the innate immunity [46]. Additionally, we assessed the haemagglutination and haemolysis titers, representing the level of natural antibodies and complement, respectively, which are important effectors of innate immunity [47,48]. Because we ran these experiments under *ad libitum* conditions we can test, if such trade-offs are also observable when resources are not directly limiting.

## Material and methods

### Experiment I

When between 50 and 70 days old, female outbred domesticated guinea pigs (*Cavia aperea* f. *porcellus*) were paired with a male (2 females to one male) and kept together in a 0.8 m^2^ enclosure. After the male was removed, the females were separated by a wire mesh, so that each one had her own enclosure of 0.4 m^2^ with contact through wire mesh to the neighbouring female. Enclosures were located in temperature controlled rooms (23°C during the day and 20°C during the night). Light was on for 14 h daily. All animals received only pellet food (Höveler, Langenfeld, Germany) and water *ad libitum*. Once per week vitamin C was added to the drinking water.

We compared the following groups:

1. Non reproducing females (n = 8) were injected subcutaneously twice with one week interval with isotonic KLH solution (400 μl containing 100 μg of KLH) on day 107 of life.
2. The corresponding control group (n = 12) was injected at the same age with the same volume of saline solution.
3. Pregnant females (n = 16) immunized on day 40 of pregnancy (the number of days into pregnancy was determined from the last day of vaginal opening) and one week later with the same amount of KLH as non reproducing females. On day 40 of their pregnancy these animals were of approximately the same age as the control animals in group 1 and 2.
4. A control group of pregnant females (n = 16) was injected twice on day 40 of pregnancy and one week later with 400 μl saline solution.
5. Lactating females (n = 10) immunized as group (1) within 48 h of parturition and again one week later.

For all groups we took blood samples and analyzed those by ELISA for specific anti-KLH antibodies (see below). Blood samples were taken weekly, 5 times beginning at the day of first injection of KLH by puncturing the marginal ear vein and collecting about 70–100 μl blood. Plasma was separated by centrifuging the blood for 10 minutes with 8000 rpm and stored at -80°C until further analysis.

### Experiment II

This experiment was run to determine, if the strong effects found in young, primiparous females (Experiment I) were generalizable to a more diverse group of animals. Females in this experiment were older and most of them had given birth before. Moreover, they received a more diverse diet throughout the experiment. Simultaneously, we wanted to determine effects on innate immunity. We always first tested for the specific anti-KLH antibody titers and only determined various aspects of the innate immunity (BKA, haemagglutination and haemolysis titers), if sufficient blood was left. This resulted in differences in sample size for the tests of adaptive and innate immunity as indicated in Table 1.

**Table 1:** Sample size for analyses of adaptive and innate immunity. Numbers in brackets indicate the number of animals for which samples were missing at different points in time. KLH: Injected with an isotonic solution of KLH. Saline: Injected with an isotonic saline solution.

	Immune system	Measured trait	State	Sample size complete (partial)
Exp. I	Adaptive immunity	anti-KLH antibody levels	Non reproducing KLH	8
			Non reproducing saline	12
			Pregnant KLH	16
			Pregnant saline	16
			Lactating KLH	10
Exp. II	Adaptive immunity	anti-KLH antibody levels	Non reproducing KLH	13
			Non reproducing saline	5
			Pregnant KLH	12
			Lactating KLH	15
	Innate immunity	BKA (%)	Non reproducing KLH	3 (2)
			Non reproducing saline	0(5)
			Pregnant KLH	10(2)
			Lactating KLH	10(5)
		Haemolysis titers	Non reproducing KLH	6(4)
			Non reproducing saline	0(5)
			Pregnant KLH	9(3)
			Lactating KLH	8(7)
		Haemagglutination titers	Non reproducing KLH	6 (3)
			Non reproducing saline	5
			Pregnant KLH	9(3)
			Lactating KLH	8 (7)

Four guinea pigs were kept together in one enclosure with a floor area of 1.6 m^2^. Enclosures were located in a room with windows to the outside at a temperature of 20 ± 2°C. Water, hay and the same guinea pig pellet food as in Experiment I were provided *ad libitum*. Fresh greens were given every other day. The drinking water was supplemented with vitamin C once a week. For breeding, a group of 3 females was kept together with one male for about one month. The non-reproducing females were kept in groups of 4.

In this experiment we compared the following groups:

1. A group (n = 5) of full grown non-reproducing females were injected with the 400 μL isotonic saline solution.
2. A group of equally full grown, non-reproducing females (n = 13) was injected with 200 μg of KLH in 400 μL of isotonic saline solution and received a boost-injection one week later.
3. Pregnant guinea pigs (n = 12) were challenged early in the second half of pregnancy (mean pregnancy day 36.7 ± 5.12 days [range 26–45] at first injection) using the same protocol and dosage as group (2).
4. Lactating females (n = 15) were also injected with 200 μg of KLH in 400 μL saline solution 1–2 days post-partum.

As in Experiment I, blood samples were collected five times beginning at the first injection on a weekly base. As stated above, blood sample volumes were not sufficient to measure the complete set of parameters for innate immunity for all females (see Table 1).

### Weight measurements

Females were weighed at the time of injection. Pups were weighed at birth and weaning (20–21 days of age). Growth of pups was measured for the period from birth to weaning (g/day).

### Immunological measurements

#### Antibody mediated adaptive immune response

To measure KLH-specific antibodies we used an enzyme-linked immunosorbent assay (ELISA) previously developed for guinea pigs [38]. The KLH antigen (Sigma, H7017) was first bound to the surface of each well of a 96-well ELISA plate (Sarstedt, 82.1581.200) when incubated overnight at 4°C. After washing the plates, to avoid unspecific binding 200 μl of bovine albumin (SERVA, 11930.02) was added to each well and incubated for one hour at 37°C. Following subsequent washing, we added 200 μl of 1:100 and 1:2000 diluted plasma samples to each well, allowing antigen-specific antibodies to bind to the antigen attached to the well when incubated for 1 h at 37°C. Samples were diluted with a washing buffer with the following composition: 8.0 g NaCl, 0.2 g KH_2_PO_4_, 1.4 g Na_2_HPO_4_ × 2H_2_O, 0.2 g KCl, 0.2 g NaN_3_ and 0.5 ml Tween diluted in 1 l distilled water at pH 7.4. After incubation, plates were washed, 1:2000 diluted goat anti-guinea pig IgG solution (SouthernBiotech, 6090–04) was added and incubated at 37°C fo 2 h. Finally, to allow detection of the magnitude of KLH-bound antibodies we added a substrate solution composed of 0.25 g phosphatase substrate (Sigma, S0942) in 100 ml buffer with 1.59 g Na_2_CO_3_, 2.9 g NaHCO_3_, 0.2 g NaN_3_, 0.12 g MgCl_2_ × 6 H_2_O in 1 L distilled water. Plates were read with 405-nm wavelength filter after 5 minutes of incubation at room temperature at darkness. All samples were run in duplicates, and the average of the two readings was our measure of antibody levels (optical density, OD) in the plasma. For the final analyses, the results of the 1:2000 dilution was used.

### Bacterial killing activity

We assessed the *in vitro* bacterial killing activity (BKA) of the plasma against *Escherichia coli* following the method of Tieleman et al. [49]. This assay has been used previously on different free-ranging and captive small mammal species, including several rodents [50–52] and is useful as it measures the functionality of the innate immunity [46]. After diluting the plasma samples 1:20 in PBS, to each diluted sample (140 μl) we added 10 μl of a suspension of live *E*. *coli* (ATCC #8739). The bacterial suspension was adjusted to a concentration of 200 colonies per 50 μl of diluted plasma-bacteria mixture. After incubation for 30 min at 37°C (mammalian body temperature), aliquots of the 50 μl of the plasma-bacteria mixture was spread onto Tryptic Soy Agar plates (#CP70.1, Carl Roth GmbH) in duplicate, and the plates were incubated overnight at 37°C. To obtain the initial number of bacteria that we had before starting to interact with the plasma, we diluted 140 μl media alone with bacterial suspension and plated immediately. On the following day the colony-forming units were counted and the bacterial killing activity (BKA%) was defined as the percent of the killed bacteria, which was calculated as 1– (average of the viable bacteria after incubation/the initial number of bacteria). The average was calculated from two plates per sample.

### Haemolysis–haemagglutination assay

We assessed the levels of natural antibodies and complement by using the haemolysis–haemagglutination assay [47] using chicken erythrocytes as target cells, both effectors being part of the innate immunity [48,53,54]. After 10 μl of plasma was twofold serially diluted in sterile PBS from 1:2 to 1:1024, we added 10 μl of 1% chicken red blood cells suspension to all wells and incubated the plates at 37°C for 90 min. After incubation, the plates were kept at room temperature and agglutination and lysis, which reflect the activity of the natural antibodies and the interaction between natural antibodies and complement [47,55], were recorded after 20 and 90 min, respectively. Titres of natural antibodies and complement were given as the log2 of the reciprocal of the highest dilution (i.e. lowest concentration) of plasma showing positive haemagglutination or lysis, respectively [47].

### Ethical note

Experiments were carried out in accordance with German animal protection law. Animal facilities are approved for keeping and breeding guinea pigs by the local government authority responsible for health, veterinary and food monitoring (Gesundheits-, Veterinär- und Lebensmittelüberwachungsamt) under the licence number 530.42 16 30–1. Manipulation of animals and blood sampling were permitted for Experiment I by the Bezirksregierung Detmold (permit 17.07.2000) and for Experiment II by LANUV, NRW, Germany (permit AK: 84–02.04.2013.A214). After the experiments the animals were kept for further research.

### Statistics

To test for differences in the response to the KLH immune challenge we built (generalized) linear mixed models separately for the two experiments using R (version 3.1.2; [56]; package lme4). All models included Individual as a random factor, reproductive state (non-reproducing control, non-reproducing—immunized, pregnant-control (Experiment I only), pregnant–immunized, lactating—immunized) as a fixed factor, the week of the sample (weeks from first injection) and the interaction between week and reproductive state. Note that week of sample was modelled as a 5-level factor for the slow, adaptive immune response because it showed a sharp, non-linear increase in Extinction after the second injection while it was modelled as a covariate for natural immunity, which showed a more linear, gradual temporal development. Comparisons were made to non-reproductive control females. When the comparison showed significant reaction differences (tested by Satterthwaite approximation in the R-package LmerTest) among reproductive states, we excluded the non-reproductive group to test for significant differences between pregnant and lactating females. Models for extinction were built assuming a Gaussian distribution. The model for bacterial killing activity also assumed a Gaussian distribution. Agglutination data were square root transformed prior to analysis to achieve a Gaussian distribution. Lysis data were strongly zero-inflated and we assumed a negative-binomial distribution. Model assumptions were validated visually using Q-Q plots.

Differences in pup growth from birth to weaning as a function of maternal reproductive state were correspondingly tested by linear mixed models taking into account litter size as fixed and maternal ID as a random factor.

## Results

### Experiment I

**Fig 1:**
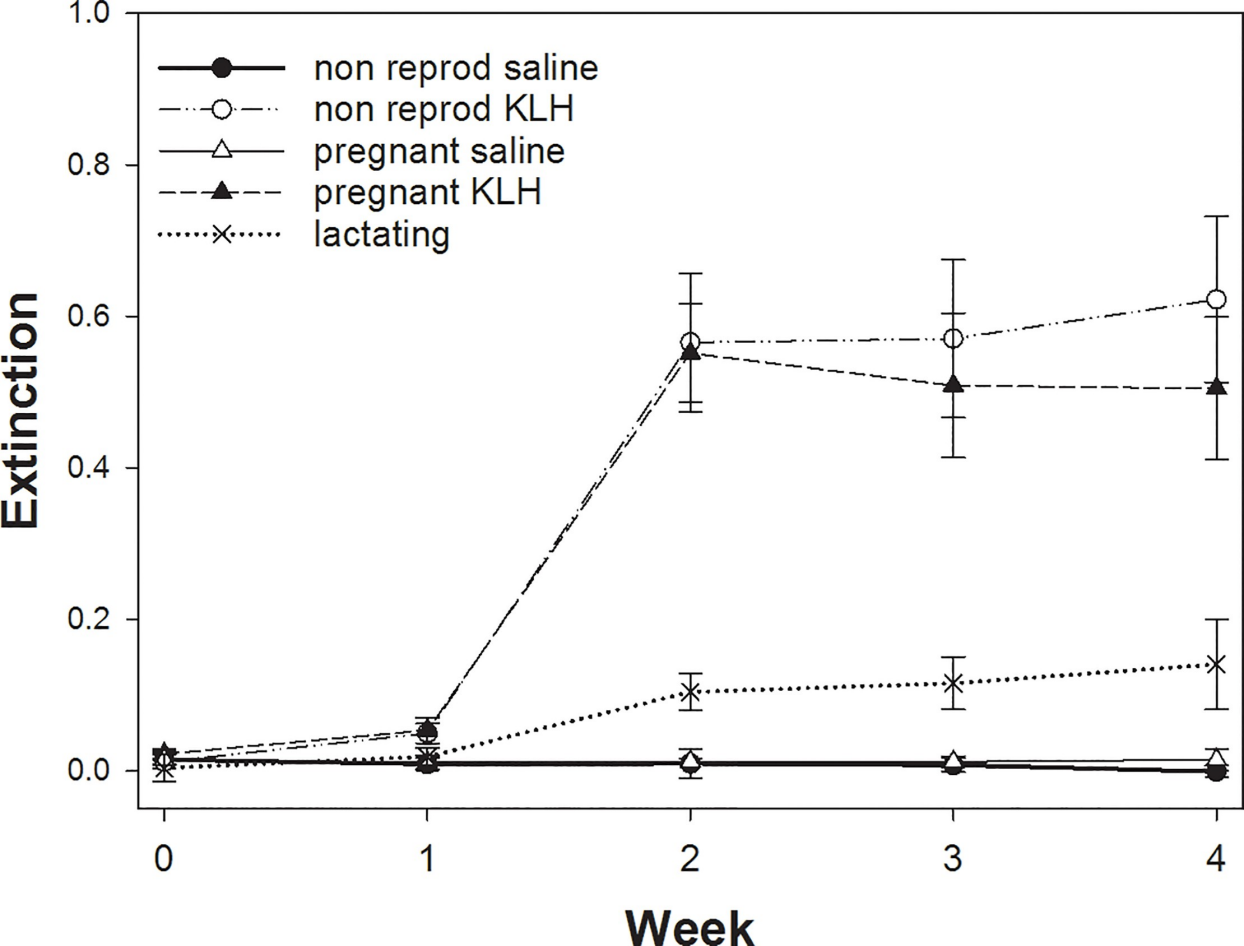
Differences in the adaptive immune response across the 4 weeks as measured by KLH-Elisa by females in Experiment I. Plotted are means and SE. For details see text.

As expected, sham injected females whether non-reproducing or pregnant showed no reaction across all 4 weeks (Fig 1). Pregnant females reacted as strongly as non-reproducing females to the KLH-challenge with a tendency to lower levels during week 4 (LMM non-reproducing versus pregnant p>0.4; only in week 4, p = 0.058). Lactating females produced significantly less antibody to KLH than the non-reproducing and pregnant females (Fig 1; LMM for weeks 2–4: non-reproducing versus lactating p<0.001; pregnant versus lactating p < 0.001).

Females in this experiment were young and primiparous. Weights for all groups are given in Table 2. Naturally, pregnant females were heavier at the start of the injections than non-reproducing or lactating females (ANOVA, p < 0.001) while non-reproducing and lactating females did not differ in body mass (ANOVA, p = 0.62; Table 2).

**Table 2:** Body mass at first injection of females and data on reproductive output in Experiment I and II. See text for statistics. KLH: Injected twice at one week interval with an isotonic solution of KLH. Saline: Injected twice at one week interval with an isotonic saline solution.

	State	Mean mass at injection ± SE	Mean litter size	Mean litter mass	Mean pup mass	Mean pup growth rate	Sample size
Exp. I	non-reproducing saline	618.0 ± 20.5	--	--	--	--	8
	non-reproducing KLH	616.1 ± 24.7	--	--	--	--	12
	pregnant KLH	784.9 ± 24.4	2.5 ± 0.19	261 ± 15.0	102.6 ± 3.9	6.21 ± 0.37	16
	pregnant saline	819.5 ± 19.8	2.4 ± 0.16	264 ± 14.0	106.0 ± 3.1	6.56 ± 0.37	16
	lactating	643.9 ± 19.1	3.0* ± 0.27	269* ± 16.7	89.7* ± 4.2	5.1* ± 0.41	10
Exp. II	non-reproducing saline	825.2 ± 34.5	--	--	--	--	6
	non-reproducing KLH	747.1 ± 26.7	--	--	--	--	7
	pregnant KLH	1154.3 ± 32.8	3.4 ± 0.37	279 ± 30.4	94.6 ± 5.5	7.61 ± 0.40	10
	lactating	866.1 ± 27.4	4.0 ± 0.17	387 ± 22.3	96.8 ± 4.7	6.98 ± 0.39	12

* Data for offspring measures based on 8 litters only.

Litter size did not differ between groups (F_2,41_ = 1.33, p = 0.28; Table 2). Taking original litter size into account, birth mass and growth rate of pups differed across the groups. Pups of non-immunized females were born heaviest (Table 2). Although the lactation group received the first injection only after the offspring were born, i.e. birth weight was uninfluenced by KLH treatment, pups of mothers immunized during lactation were lighter at birth (on average 15 g, t = -4.8, p < 0.001). Also, taking litter size into account, pups of mothers immunized during pregnancy weighed less than pups of non-immunized mothers (t = -1.9, p = 0.05), but more than pups of mothers immunized during lactation (t = 3.2, p = 0.004; Table 2).

Growth rate of pups whose mothers were immunized during lactation was significantly lower than growth rate of pups from non-immunized mothers (t = -1.45, p = 0.03), while growth rate of pups whose mothers were immunized during pregnancy did not differ from offspring of females that had only received a saline injection (Table 2; t = 0.17, p = 0.57). Pup growth rate of the pregnancy group was higher than that of pups of the lactation group (t = 1.39, p = 0.03; see Table 2).

### Experiment II

**Fig 2:**
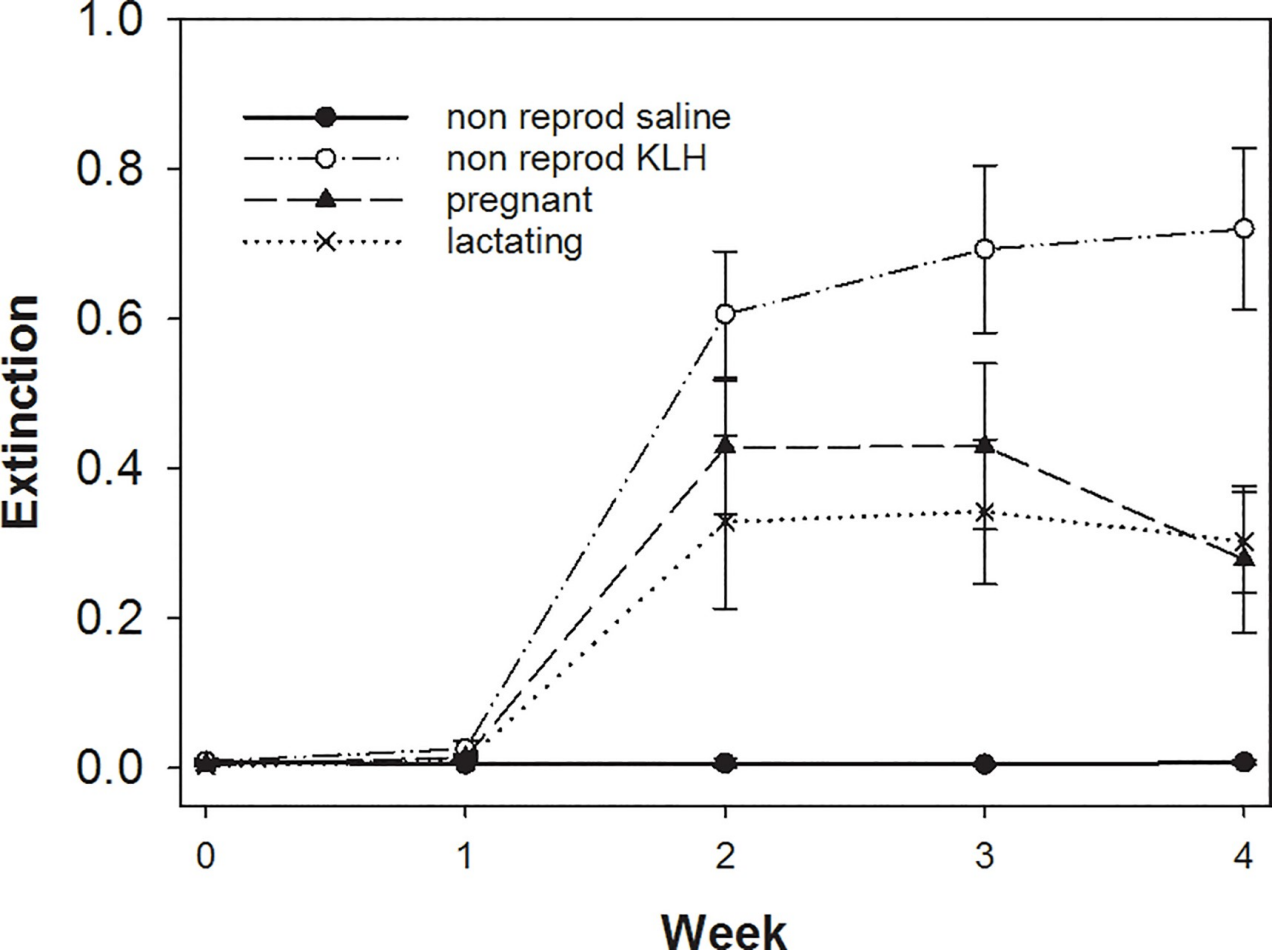
Differences in adaptive immune response across the 4 weeks measured by KLH-Elisa by females in Experiment II. Plotted are means and SE. For details see text.

Pregnant and lactating females showed significantly lower responses to the KLH-challenge than non-reproducing females (Fig 2; LMM pregnant versus non-reproducing: week 2 p < 0.05; week 3 p < 0.01; week 4 p < 0.001). We found only minor, non-significant differences in antibody concentrations between pregnant and lactating females (Fig 2; LMM p > 0.05).

**Fig 3:**
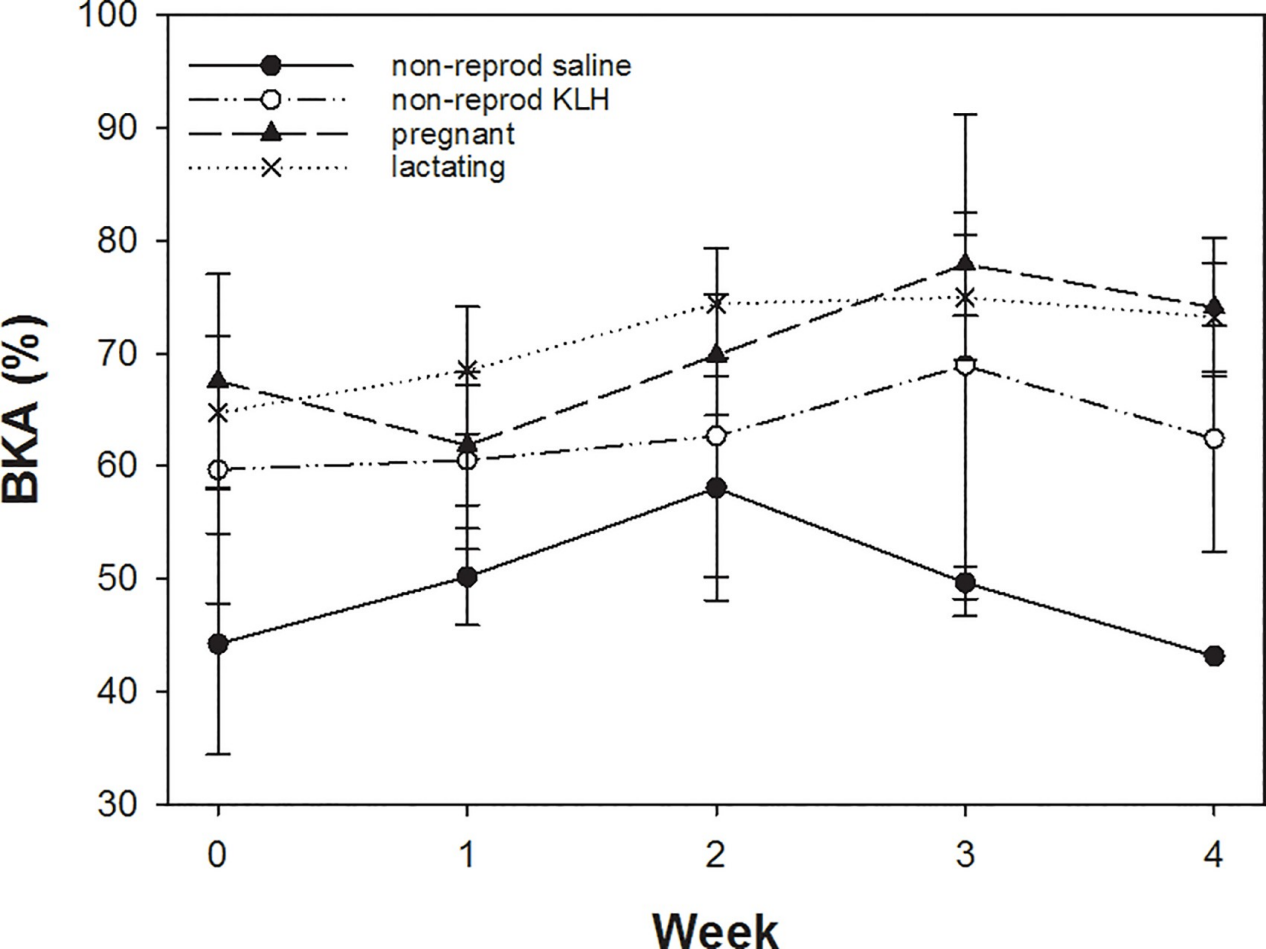
Differences in BKA across the 4 weeks for control, non-reproducing, pregnant and lactating females. Plotted are means and SE. For details see text.

BKA values (Fig 3) did not differ between non-reproducing saline and KLH-injected females (t = 1.6, p = 0.11). Pregnant and lactating females showed higher levels than non-reproducing females (pregnant: t = 4.1, p < 0.001; lactating: t = 4.5, p < 0.001), but pregnant and lactating females never differed in the expressed levels (t = -0.39, p = 0.7). In all four groups, the response level appeared to increase with time (LMM, t = 4.3, p < 0.001).

**Fig 4:**
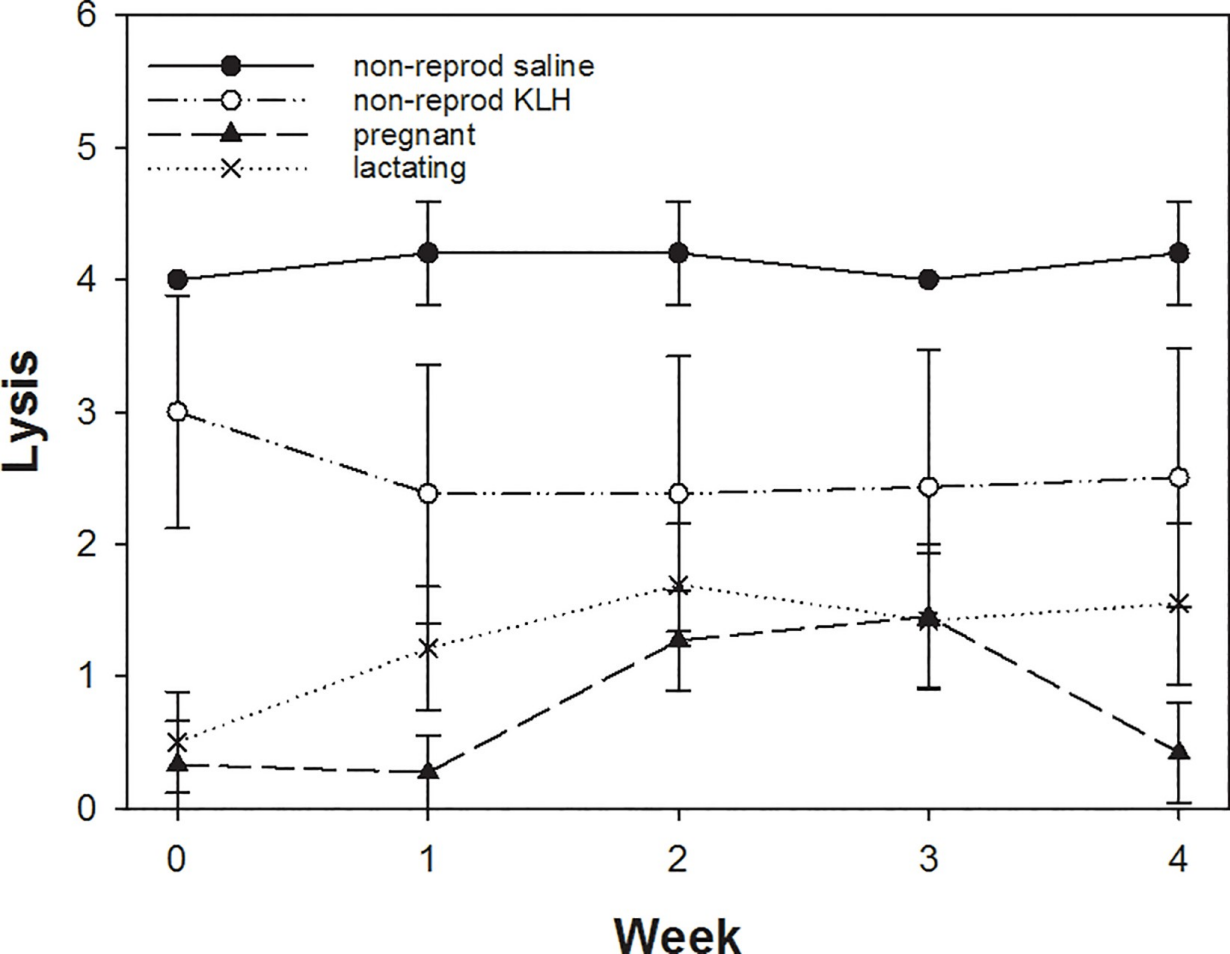
Differences in haemolysis titers across the 4 weeks for non-reproducing, pregnant and lactating females. Plotted are means and SE. For details see text.

The haemolysis titers (Fig 4) did not change over time (z = 1.4, p = 0.16). Compared to the non-reproductive saline injected females all groups of KLH-challenged females showed slightly reduced haemolysis titers (z = -3.3, p = 0.009). Pregnant and lactating females displayed even lower haemolysis titers compared to the two non-reproductive groups (pregnant: z = -9.1, p < 0.001; lactating: z = -7.7, p < 0.001) and the haemolysis titers of pregnant females were even further reduced in comparison to lactating females (z = -2.8, p = 0.005).

**Fig 5:**
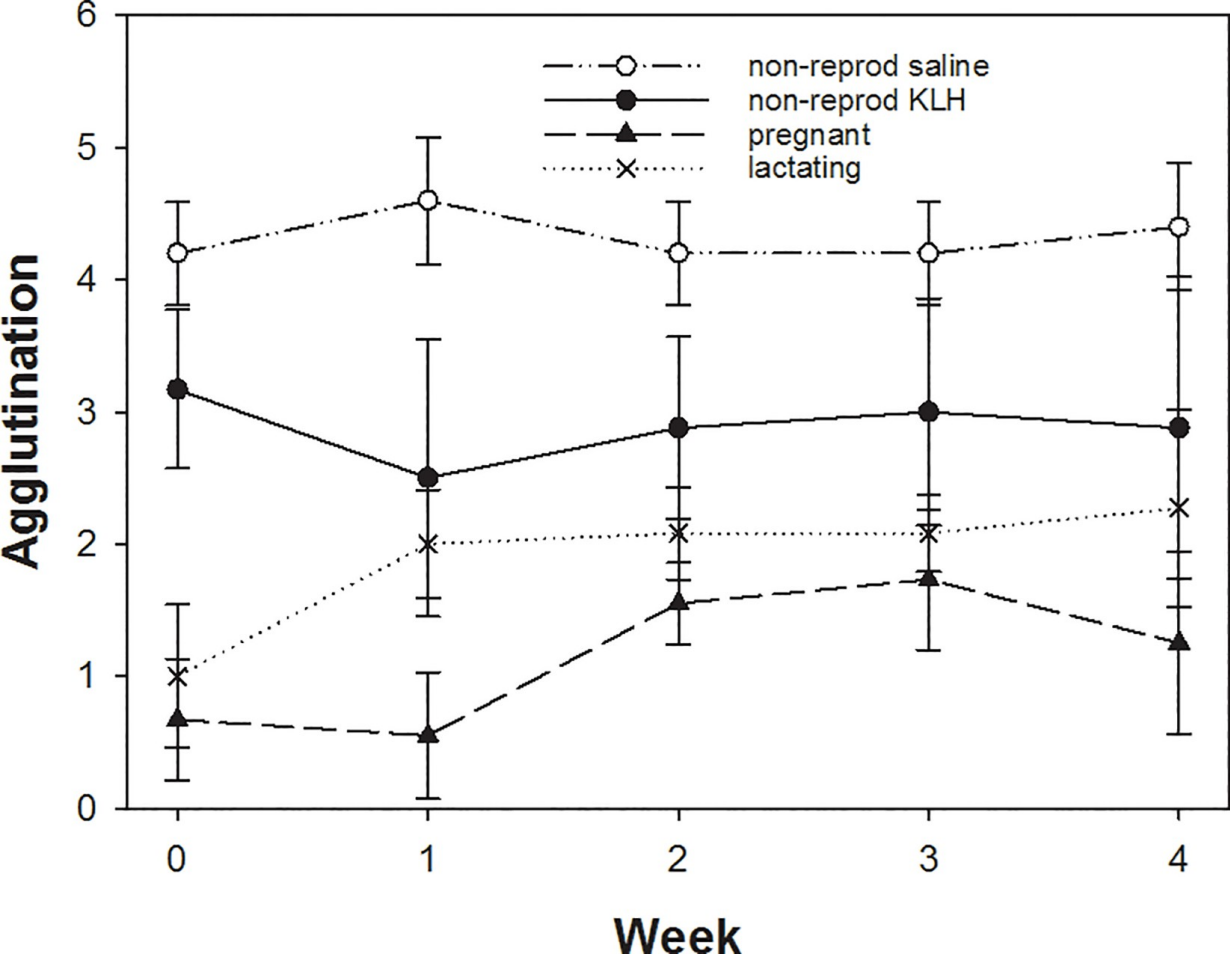
Differences in haemagglutination titers across the 4 weeks for non-reproducing, pregnant and lactating females. Plotted are means and SE. For details see text.

The haemagglutination titers of all groups differed significantly from each other (Fig 5). On average, non-reproducing saline-injected females had higher haemagglutination titers than all other groups followed by the non-reproductive KLH-injected group (t = 4.4, p < 0.001). Lactating females had significantly lower haemagglutination titers than the non-reproductive groups but higher titers than pregnant females (t = 2.6, p = 0.03). The temporal development differed between reproducing and non-reproducing females. There was no change over time for the saline-injected (t = 0, p = 1) and the KLH-injected non-reproductive groups (t = 0.34, p = 0.74), whereas for both, lactating (t = 2.2, p = 0.03) and tendencially the pregnant group (t = 1.86, p = 0.06), haemagglutination titers increased over time.

At the time of first injection, females of all treatment groups in this experiment were heavier than in Experiment I (t-tests, all p < 0.001; Table 2). Again, pregnant females represented the heaviest group at first injection (ANOVA, p < 0.001). Lactating females were slightly heavier than non-reproducing females (t = 2.3, p = 0.03; Table 2). Average litter size of the females of the pregnant and of the lactation group did not differ significantly (t = 2.13, p = 0.16; Table 2). Contrary to Experiment I, mass at birth did not differ between the pregnant and lactation group (LMM pregnant versus lactating mothers, t = -0.69, p = 0.50). However, similar to Experiment I and taking litter size into account, growth rate of pups (from birth to weaning; Table 2) was higher in litters of the pregnancy group than in those of the lactation group (LMM, t = 2.5, p = 0.02 Satterthwaite approximation).

## Discussion

Reproducing females reacted less to a KLH challenge and showed lower lysis and agglutination than non-reproducing females. This confirms our expectation that increased resource demands during reproduction will decrease the immune response. We found mixed evidence that due to higher energy demand this reduction was more strongly expressed during lactation than during pregnancy. Nevertheless, females immunized during lactation seemed strongly affected as their pups suffered reduced growth rates in comparison to females immunized during pregnancy. We also found different responses within the innate immune system. BKA was increased in the reproducing animals beyond that in non-reproducing ones whereas haemagglutination and haemolysis titers were reduced. Thus we observed no simple trade-off between reproductive effort and the immune activity nor between the adaptive and innate immune responses, but a potentially adaptive shift in activity within the natural system. It is noteworthy that these reactions happened despite the fact that the animals had continuous *ad libitum* access to food. This supports our expectation that some changes in the immune system might be due to physiological regulation which produces a predictive response largely independent of resource limitation.

### Effects of reproduction on antibody-mediated, adaptive immunity

KLH is known as an immuno-stimulant that does not cause illness or fever, but reliably induces an immune response and temporarily increases the metabolic rate [57,58]. As predicted, reproduction reduced the specific immune response, particularly of the lactating females whether young as in Experiment I or fully grown as in Experiment II. In Experiment I, lactating females showed an intermediate response, constantly lower than pregnant females but elevated above control values from the second week onwards, while in Experiment II lactating females showed an elevated response which did not differ from that of pregnant females. Hence, the younger females of Experiment I apparently invested less in the adaptive immune response than the older ones of Experiment II. This could be explained by differences in protein demand between the groups. Young guinea pig females still grow during pregnancy [36]. Consequently, young females will have a higher protein demand than fully grown ones. This would agree with our finding that pregnant females in Experiment I reacted with a lower immune response to the KLH challenge.

Energy and protein demand is still higher in lactating than in pregnant females [41] and even under *ad libitum* conditions may be hard to cover while exporting energy and protein through milk. In addition, the animals in Experiment I received only pellet food, but no hay or fresh food in comparison to the animals in Experiment II, which constitutes reduced nutritional quality. This may explain the particularly low response to the KLH challenge of lactating females in Experiment I. Further experiments are needed to prove which of these factors led to the low KLH response of lactating females in Experiment I.

Similar effects have been shown in other species of mammals. Sheep were shown to come close to a peri-parturient breakdown of their immune defense against an abomasal nematode, apparently prioritizing reproductive function over defense against this parasite [25]. Strains of sheep selected for resistance to nematode infection lost this resistance during lactation [59] and, similarly, Festa-Bianchet [24] found that lactating bighorn sheep (*Ovis canadensis*) ewes had greater fecal counts of lung-worm larvae than non-lactating females.

### Effects of reproduction on innate immunity

We assessed the *in vitro* bacterial killing activity (BKA) of the plasma against *Escherichia coli*, a functional method that measures the overall functionality of the innate immune response independently of individuals’ pathogen exposure history [46,52,60]. Additionally, we measured two important effectors of the humoral innate immunity, the levels of natural antibodies and complement, via assessing the haemagglutination and the haemolysis titers [47,61]. We expected to see a trade-off between adaptive and innate immunity. However, our findings show a more complex response. Reproducing and non-reproductive females showed clear differences within aspects of the innate immune system. BKA was higher in reproducing than non-reproducing females, which showed lower haemagglutination and haemolysis titers. Thus, there was no simple trade-off between the adaptive and the innate immune system. The increased BKA level of pregnant and lactating females may reflect the need for protection against bacterial infection in a species with post-partum mating. Post-partum mating creates a situation where through copulation bacteria may be introduced into the uterus that still presents fresh placental scars as a potential wide open entrance for bacteria. Stimulation of the specific immune response by KLH appeared to reduce the haemolysis-haemagglutination titers in non-reproducing females, but to increase the BKA. Taken together, these results suggest a complex pattern of interaction between the innate and the adaptive immune system. Besides these results, the outcome of our experiment is also intriguing since previously it has been shown in birds and bats, that the BKA is mainly mediated by complement [62]. Thus, it could have been plausible to expect to have similar results for BKA and haemolysis titers, though in other species different outcome have been previously described [48]. Further studies are required to understand which humoral effectors are responsible for the increase in BKA in the reproducing female guinea pigs.

### Effects of immunization on offspring development

The lower pup birth mass of females in Experiment I immunized during pregnancy in comparison to control indicates the allocation of resources (energy, protein) to the immune response while gestating a litter. It is uncertain how to interpret the different growth rates of pups of the pregnancy and lactation groups in Experiment I as one could argue that the low growth rate of pups in the lactation group in Experiment I was related to their low birth mass. However, in Experiment II the birth mass of pups did not differ between the pregnancy and lactation group, but the growth rate of pups of the lactation group was lower, suggesting that investment into the adaptive immune response did cause a reduced allocation to offspring in the lactation group. These observations suggest that the allocation of resources to the immune response during pregnancy as well as during lactation led to reduced allocation to offspring even under *ad libitum* food conditions. In other words, the adaptive immune response induces a trade-off between maintenance of maternal condition at the expense of offspring development as similarly observed in ageing female wild cavies (*Cavia aperea*) [63] and other animals [64,65].

### Evidence for a trade-off or obligate response to reproduction?

Discrepancies between studies investigating trade-offs between reproduction and the immune system may result from both, difference in resource abundance and from differences in the assessed immune parameters [8]. French et al. [14] suggested, similar to Tuomi et al. [66], that trade-offs may become manifest only under conditions of resource shortage. On the other hand, they also pointed out that animals may have evolved predictive physiological responses that allow them to adjust the allocation of energy to foreseeable changes in energy demand of different functions.

There is substantial evidence from domestic species that breeding for high production will reduce immune function. For example, in dairy cattle there is a negative correlation between milk yield and health [8]. As such trade-offs are heritable (for review see [8]) this suggests that the observed trade-off is a regulated response partly independent of resource availability [67]. This is to be expected if organisms have evolved priorities allowing them to allocate resources to various functions in a hierarchical fashion [68]. Evolutionary adaptations, for example, allow organisms to predict seasonal abundance of resources. Guenther et al. [54] found strong effects of the season of birth on innate and adaptive immune effectors in the cavy (*Cavia aperea*) which is the wild ancestor of the guinea pig. This suggests that organisms indeed are “informed resource users” [68], i.e. animal physiology is geared to predict resource shortages or times of plenty and adjust accordingly. This has often been documented with respect to seasonal changes [69], but it may equally apply to the changes involved in the predictable physiological needs during reproduction. This control of homeostasis has been called homeorhesis, which represents “the orchestrated or coordinated changes in metabolism of body tissues to support a physiological state” [70]. Homeorhesis was indeed initially described for the state of lactation where marked alterations in the partitioning of nutrients and metabolism occur to accommodate the export of energy and nutrients through the activity of the mammary gland. Our results demonstrate that such allocation shifts occur also under *ad libitum* conditions and involve complex changes even within the immune system.
